# Editorial: A year in review: discussions in systems endocrinology

**DOI:** 10.3389/fendo.2023.1236837

**Published:** 2023-09-01

**Authors:** Óscar Lorenzo, Animesh Acharjee

**Affiliations:** ^1^ Laboratory of Diabetes and Vascular Pathology, IIS-Fundación Jiménez Díaz, Medicine Department, Universidad Autónoma, Madrid, Spain; ^2^ Spanish Biomedical Research Centre on Diabetes and Associated Metabolic Disorders (CIBERDEM) Network, Madrid, Spain; ^3^ Institute of Cancer & Genomic Sciences, University of Birmingham, Birmingham, United Kingdom; ^4^ Institute of Translational Medicine, Birmingham, United Kingdom

**Keywords:** systems endocrinology, biomarker, therapeutics, diabetic nephropathy, diagnosis

Endocrine systems, also referred to as hormone systems, are found in all mammals and many other species. Endocrine systems are made up of glands all over the body; hormones are made by these glands and released into the bloodstream or fluid around the cells and receptors in different organs and tissues recognize the hormones and react to them. Importantly, inter-tissue communication via secreted proteins has been established as a vital mechanism for physiologic homeostasis and pathological development.

This editorial summarizes the contributions to the Research Topic in Frontiers in Endocrinology, titled “*A year in review: Discussions in Systems Endocrinology*” that ran from May 2022 to May 2023. This Research Topic was launched with the aim of providing a platform for researchers working in the Endocrinology field to share their recent advances on endocrine-related pathologies and potential new targets for therapy and diagnosis. A total of four original articles were selected for publication among the submissions received. A summary of each manuscript is detailed below.

During the COVID-19 pandemic, timely and appropriate vaccination was key to reduce morbidity and mortality. The effects of COVID-19 vaccination were influenced by patients’ health status and involved systemic physiological reactions (1). Thus, endocrine dysfunction after COVID-19 vaccination has raised clinical concerns, ranging from pituitary apoplexy, thyroid dysfunction, hyperglycemia and diabetes, and adrenal insufficiency to hypogonadism (2). In this regard, Pezzaioli et al. described in a narrative review the current knowledge on the potential endocrine adverse effects of COVID-19 vaccination. Despite social alarm and two years of COVID-19 vaccines, the available data showed that endocrine side effects are generally rare. Among them, thyroid disorders are the most common, for example, subacute thyroiditis and Graves’ disease. Diagnosis of type-1 diabetes mellitus has also been unusual and adrenal and pituitary events even anecdotal. Moreover, COVID-19 vaccines have not impacted on the female reproductive system or on male and couple fertility. Therefore, the endocrine system may not be heavily threatened by the current COVID-19 vaccinations. Nevertheless, the specific endocrine phenotype and the endocrine interactions in each patient might favor future secondary effects.

In addition, these endocrine interactions can be essential for type-I diabetes progression. The genetic background determines type-I diabetes predisposition; however, the autoimmune process against β-cells can be also influenced by environmental triggers such as endocrine-disrupting chemicals (3). Using bioinformatics, researchers have integrated global multi-tissue expression data and publicly available resources to identify and functionally annotate novel circuits of tissue-tissue communication (4). Yang et al. analyzed the quantitative impact of various hormonal disorders on glucose imbalance, advancing the precision treatment of secondary diabetes. Excessive glucocorticoids, epinephrine, and growth hormone could trigger dysglycemia. The authors performed extensive differential equation–based modelling to understand the impact of hyperthyroidism on the progression of diabetes. Furthermore, model simulations indicated that timely thyroid treatment could halt hyperglycemia progression and prevent beta-cell failure. Thus, diagnosis of hormonal disorders together with blood sugar tests may be useful as significant measures for the early detection and treatment of diabetes.

Both type-I and type-II diabetes can seriously damage the kidney and renal function. One out of three adults with diabetes have kidney disease, and diabetic nephropathy can reduce the kidneys’ ability to remove waste products and extra fluid from the body. However, its pathogenesis remains elusive and current therapies are only modestly effective. Interestingly, some researchers have analyzed the genome-wide expression to identify pathways involved in diabetic nephropathy. The authors used four publicly available expression datasets from the Gene Expression Omnibus (GEO) and identified differentially expressed genes (DEGs). Those genes were further clustered in different modules and hubs for construction of molecular networks. Metabolic processes, cell cycle control, and apoptosis were among the top enriched pathways (5). In this sense, Li et al. found 55 DEGs in diabetic nephropathy, including 38 upregulated and 17 downregulated genes. The fibronectin-1 (*FN1*) gene was correlated with four genes (*COL6A3*, *COL1A2*, *THBS2*, and *CD44*) and with the development of diabetic nephropathy through the extracellular matrix-receptor interaction pathway. Thus, *THBS2*, *COL1A2*, *COL6A3*, and *CD44* may be novel biomarkers and candidates for targeted therapeutics for this disease.

25-hydroxyvitamin-D deficiency is a common disorder in diabetic patients and may be a risk factor for diabetic nephropathy. Likely, the link between 25-hydroxyvitamin-D and inflammation, oxidative stress, and extracellular matrix accumulation may underlie the activated molecular mechanisms (6). Similarly, higher levels of serum uric acid have been associated with an increased risk of diabetic nephropathy in patients with type-1 and type-2 diabetes (7). However, the causality and direction of the association between 25-hydroxyvitamin-D and uric acid have not been established. In this regard, using two-sample bidirectional mendelian randomization analysis, Han et al. suggested a causal link between both uric acid and 25-hydroxyvitamin-D. To achieve this, the authors used Global Urate Genetics Consortium GWAS databases comprised of large populations of European ancestry that contained 49 cohorts from multiple countries. A 1 mg/dl increase in uric acid was associated with a 0.74 nmol/L decrease in 25-hydroxyvitamin-D. No causal relationship was, however, found the opposite way. Thus, increased levels of uric acid should be considered in diabetic patients with vitamin D deficiency.

This editorial provides an overview of a recent Frontiers in Endocrinology Research Topic that discussed novel therapeutic approaches and new diagnosis tools for endocrine-related injuries such as those occurring after COVID-19 or diabetes.

## Author contributions

All authors listed have made a substantial, direct, and intellectual contribution to the work and approved it for publication.
